# Supplementary figures and images for: CLIPPER 2.0: Peptide-Level Annotation and Data Analysis for Positional Proteomics
Source: Mol Cell Proteomics. 2024 May 3;23(6):100781. doi: 10.1016/j.mcpro.2024.100781 (PMC11192779; doi:10.1016/j.mcpro.2024.100781)

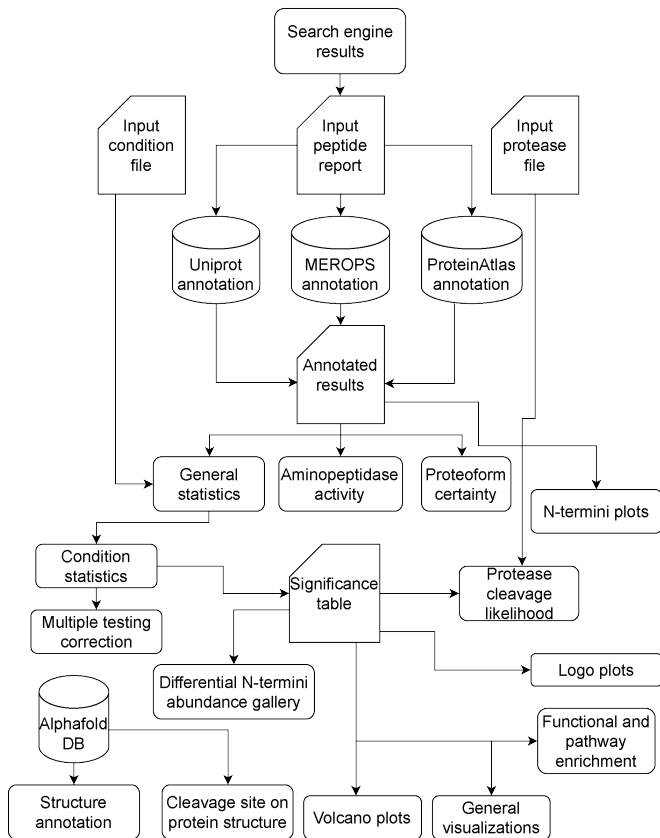

Supplement: Supplemental Figure S1 — CLIPPER 2.0 flowchart indicating different features, capabilities and results generated. [file mmc1.pdf]

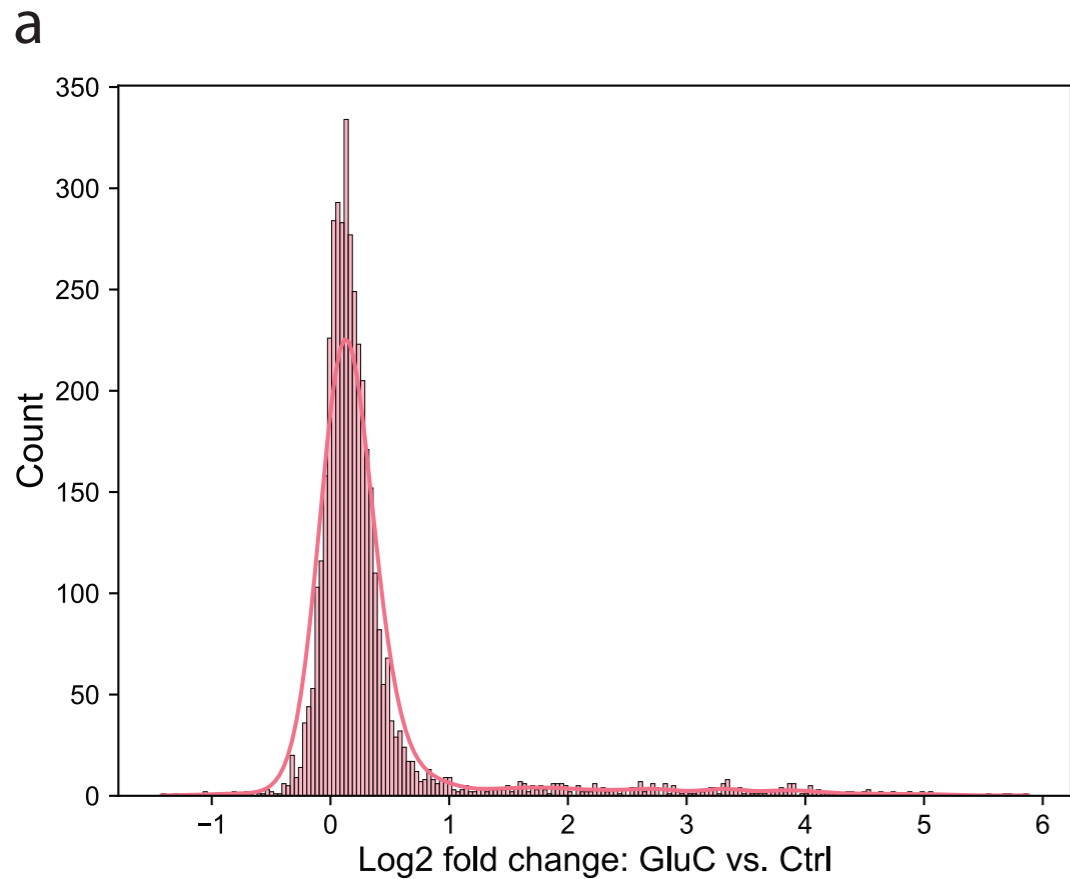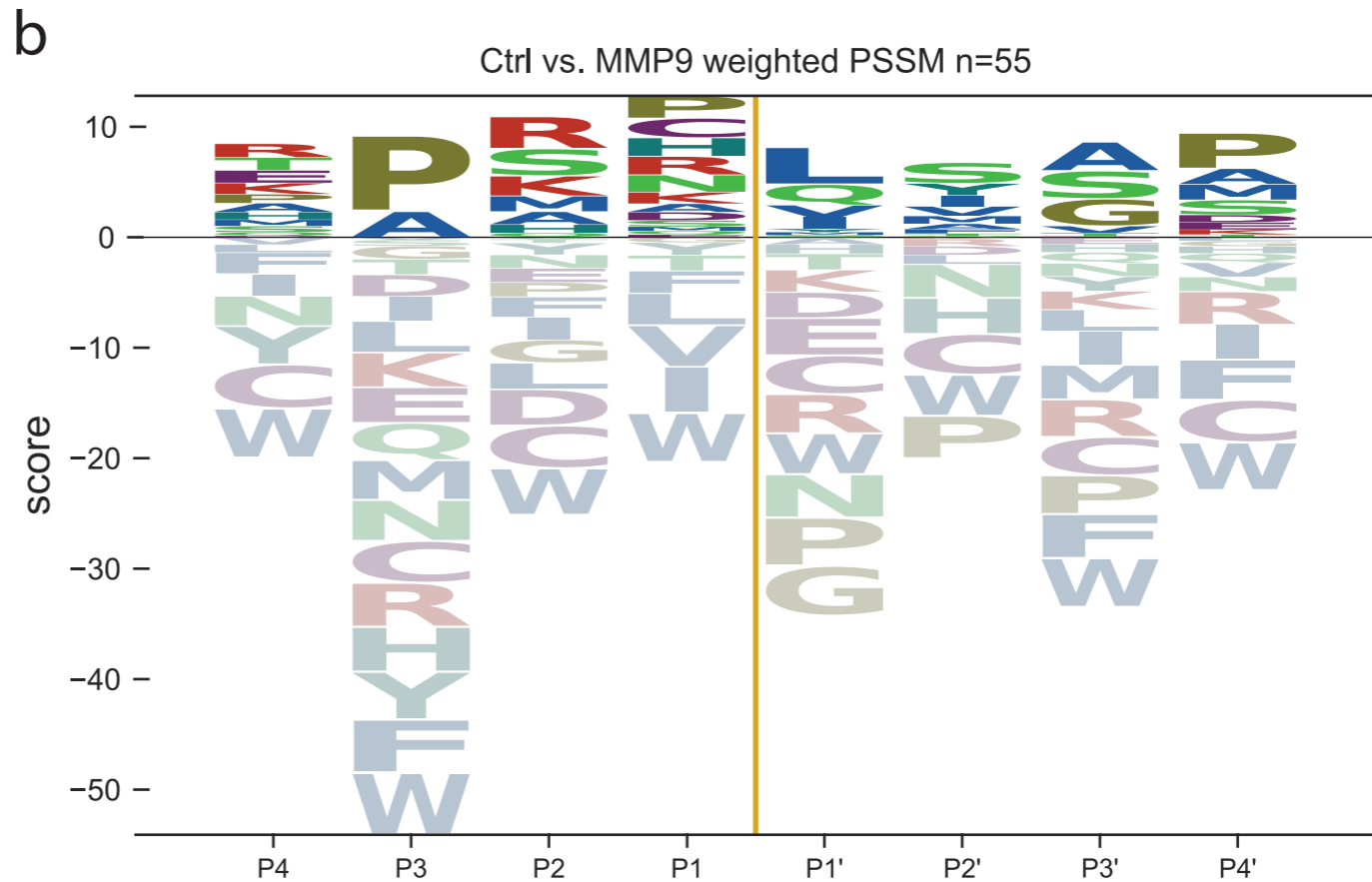

Supplement: Supplemental Figure S2 — A, fold change histogram distribution with KDE estimation. B, PSSM values for MMP9 specificity visualized as a cleavage logo. [file mmc2.pdf]

Mean values per condition

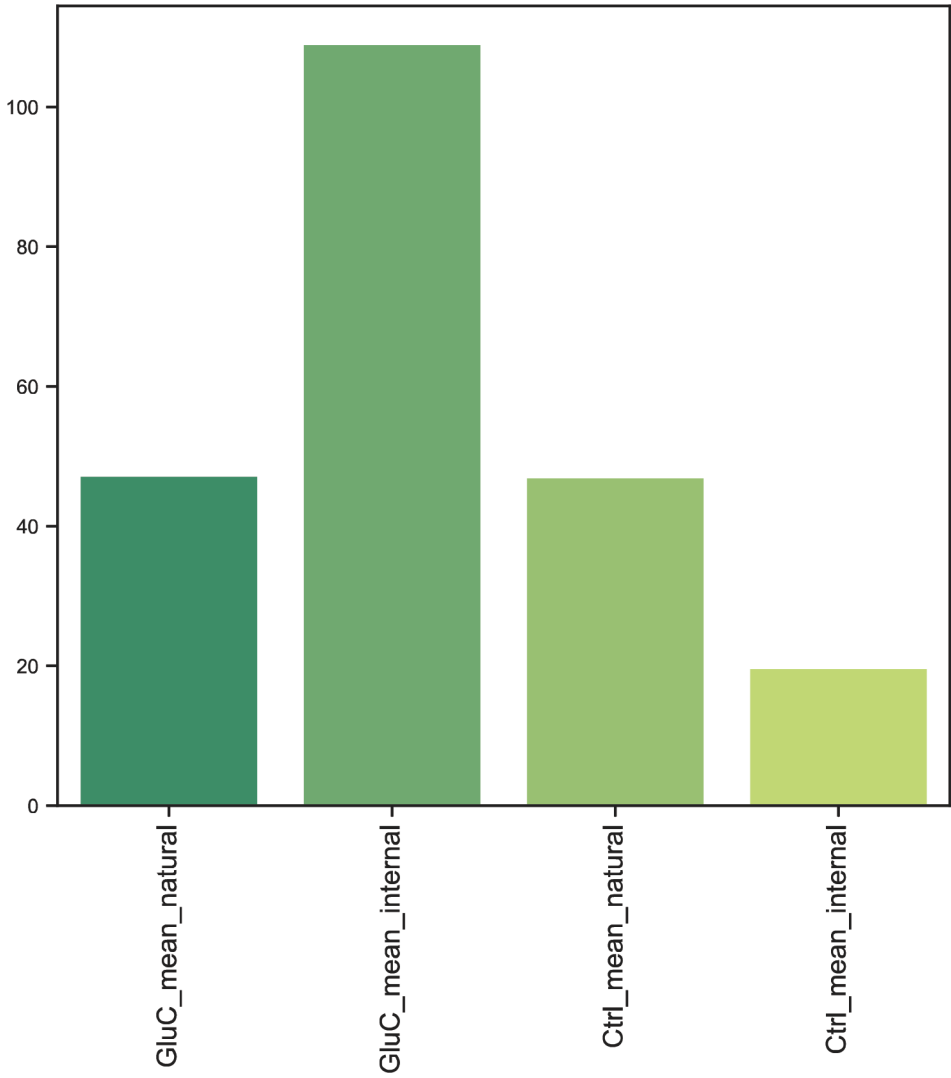

Peptides with quant values

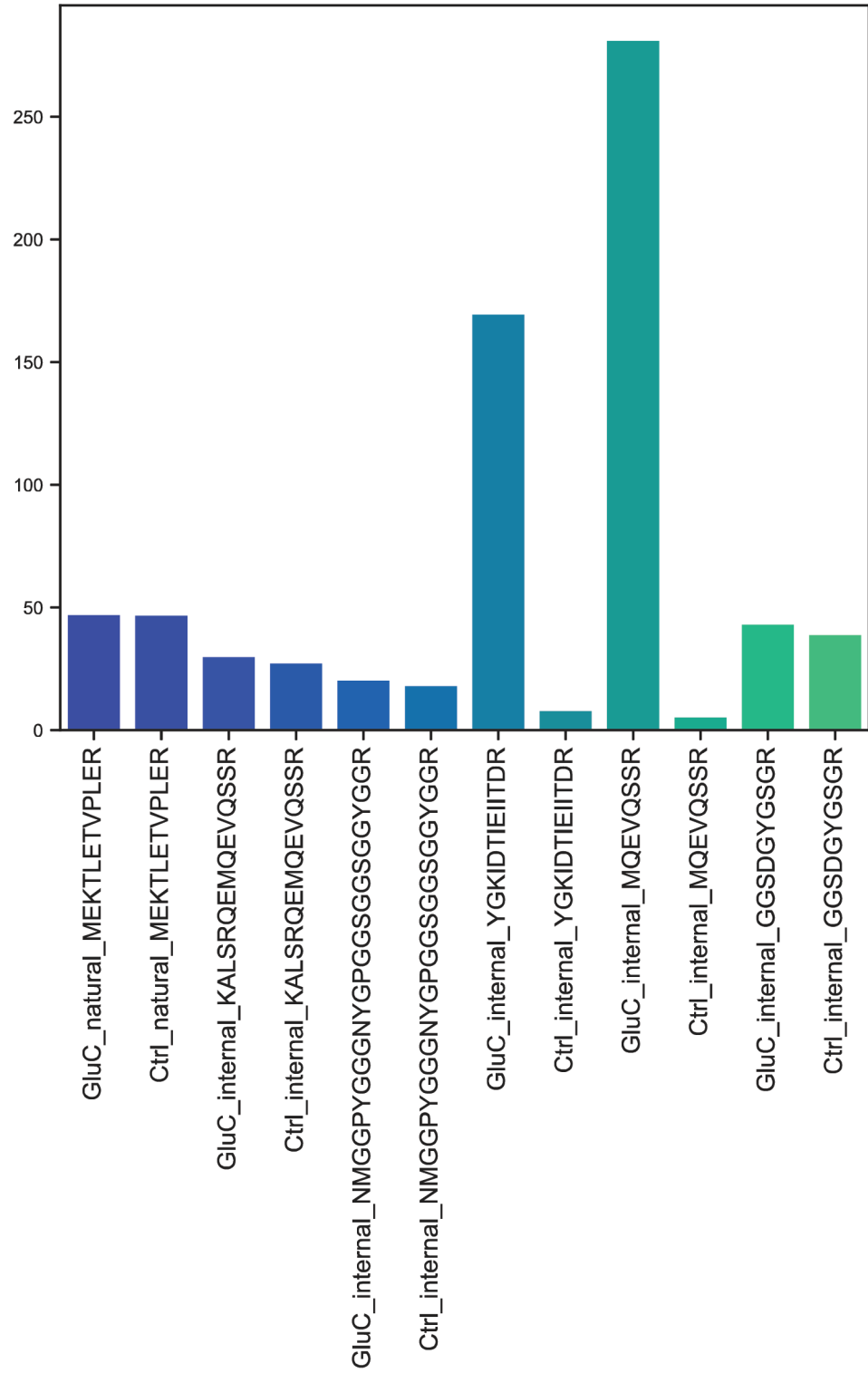

Supplement: Supplemental Figure S3 — Naturaland internal peptide abundance for termini identified in HNRNPA2B1 (Uniprot ID: P22626) as a mean abundance (left), and for each individual peptide (right). [file mmc3.pdf]

**a**

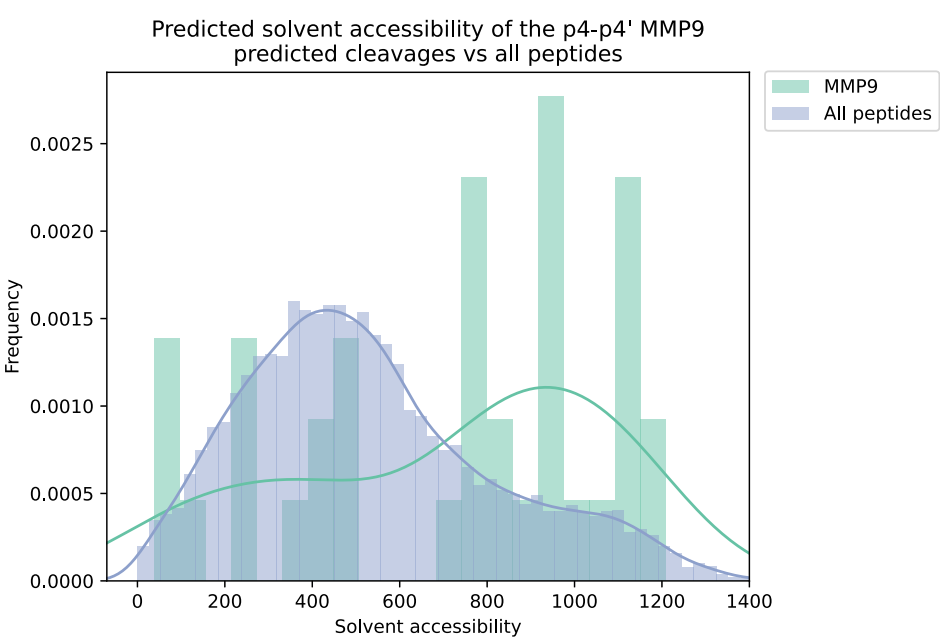

**b**

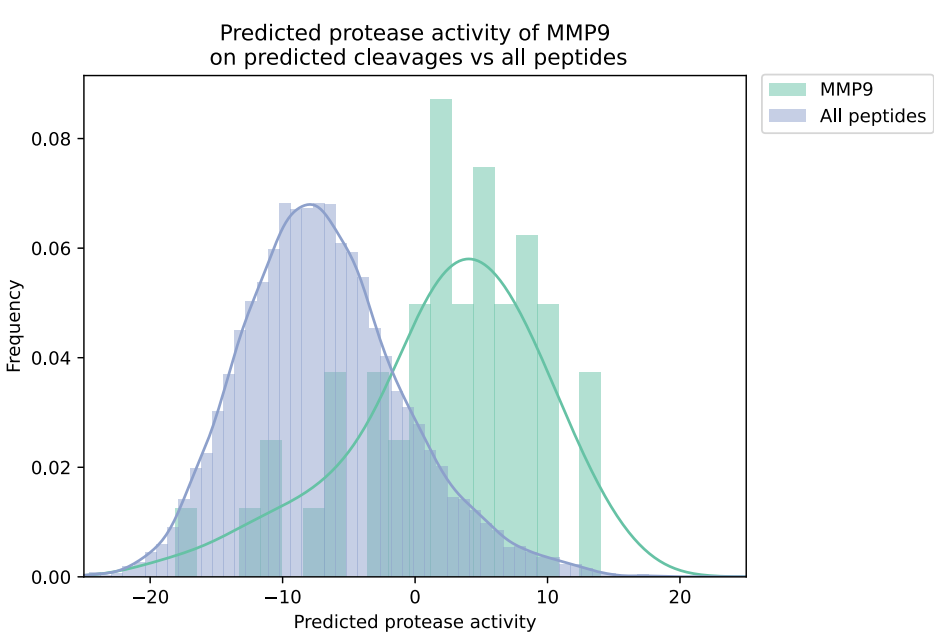

**c**

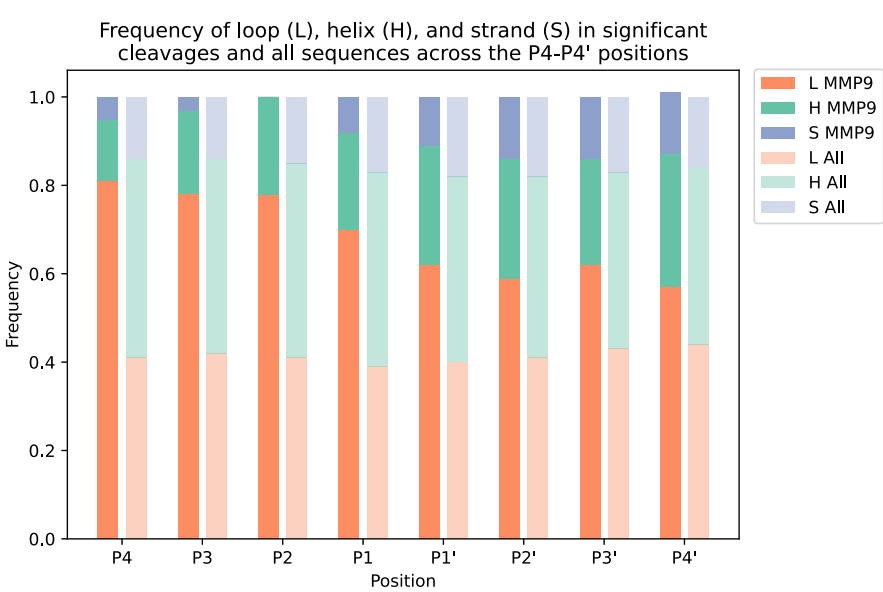

Supplement: Supplemental Figure S4 — Comparisons of feature prediction distributions between internal quantified peptides which are statistically higher in abundance in MMP9 treated native lysates (MMP9) and all quantified internal peptides (All peptides).A, distributions of predicted solvent accessibility in the p4-p4′ cleavage region. B, distributions of protease prediction scores generated with a PSSM based on known MEROPS cleavages. C, frequencies of predicted secondary structures in p4-p4′ cleavage region using AlphaFold models and PyMol. [file mmc4.pdf]
